# Psychological and Coping Strategies Related to Home Isolation and Social Distancing in Children and Adolescents During the COVID-19 Pandemic: Cross-sectional Study

**DOI:** 10.2196/24760

**Published:** 2021-04-27

**Authors:** Abduljaleel Abdullatif Zainel, Hamda Qotba, Alyaa Al-Maadeed, Sadriya Al-Kohji, Hanan Al Mujalli, Atif Ali, Lolwa Al Mannai, Aisha Aladab, Hamda AlSaadi, Khalid Ali AlKarbi, Tholfakhar Al-Baghdadi

**Affiliations:** 1 Primary Health Care Corporation Doha Qatar; 2 Hamad Medical Corporation Doha Qatar

**Keywords:** COVID-19, coronavirus, pandemic, psychological, coping strategies, children, adolescents, Qatar

## Abstract

**Background:**

In December 2019, a novel coronavirus called SARS-CoV-2 was identified as the cause of a cluster of pneumonia cases in Wuhan, China. It rapidly spread due to human-to-human transmission, resulting in a global pandemic. Nearly every country, including Qatar, has established guidelines and regulations to limit the spread of the virus and to preserve public health. However, these procedures have been associated with negative effects on the psychological and intellectual well-being of individuals, including children and adolescents.

**Objective:**

The objective of this study was to determine the psychological influence of home isolation and social distancing on children and adolescents during the COVID-19 pandemic in Qatar, and the strategies used to cope with these measures.

**Methods:**

This cross-sectional study was undertaken using an online questionnaire administered through SMS text messaging. All home-isolated children and adolescents registered at the Primary Health Care Corporation aged 7-18 years were invited to participate in the study. Children and adolescents with intellectual disadvantages were excluded. A *P* value of .05 (two-tailed) was considered statistically significant.

**Results:**

Data were collected from 6608 participants from June 23 to July 18, 2020. Nearly all participants adhered to the official regulations during the period of home isolation and social distancing; however, 69.1% (n=4568) of parents believed their children were vulnerable to the virus compared to 25% (n=1652) who expressed they were not vulnerable at all. Higher levels of anger, depression, and general anxiety were prevalent among 1.3% (n=84), 3.9% (n=260), and 1.6% (n=104) of participants, respectively. The mean score for the emotional constructs anger and depression decreased with increased compliance with regulations (*P*=.04 and *P*=.11, respectively). The differences in mean score for all psychological and coping strategies used among participants across the 3 levels of vulnerability to SARS-CoV-2 were statistically significant. The mean score varied little with increasing reported vulnerability to the virus. This mild variation can make a difference when the sample size is large, as is the case in this study.

**Conclusions:**

Screening for psychological and social disruptions is important for the development of strategies by schools and health care providers to assess and monitor behavioral changes and negative psychological impact during post–COVID-19 reintegration. Participants experiencing higher levels of anxiety should be given more attention during reintegration and transitional phases in schools. Although electronic devices and social media platforms may have lowered anxiety levels in some cases, it is important to address how they are used and how content is tailored to children and adolescents. It is also important to maintain an active lifestyle for children and young persons, and encourage them not to neglect their physical health, as this promotes a better psychological state of mind.

## Introduction

Coronaviruses are pathogens of humans and animals. In December 2019, a novel coronavirus was identified as the cause of a cluster of pneumonia cases in Wuhan, Hubei Province, China. It spread rapidly, resulting in an epidemic throughout China, followed by an increasing number of cases in other countries due to human-to-human transmission and leading to a global pandemic. In February 2020, the World Health Organization named the disease COVID-19. This pandemic affected almost all members of societies in all countries around the world, including the elderly and children. Fortunately, the rate of vulnerability and cases is lower among children [1,2].

The pandemic caused devastation across the globe, affected the daily lives of millions, and left a deep mark on people’s minds. However, historically during difficult times such as wars, humans have exhibited resiliency over time to cope with the effects of such tragedies. In recent times, social media has played a key role in enabling both our interconnectedness with family, friends, and colleagues, as well as a less-welcomed 24-hour reinforcement of our anxieties [3].

Almost all countries around the world, including Qatar, have established guidelines and regulations for the public to follow, in order to limit the spread of the virus and to preserve the health of community members and families. These regulations and instructions focused not only on closing schools and outdoor activities but also imposed measures like homeschooling, home isolation, social distancing, and quarantine. Unfortunately, these procedures are often associated with some negative effects on the psychological and intellectual well-being of people, including children and adolescents [4,5]. These negative psychological and intellectual effects among children include confusion, stress, anger, detachment from others, irritability, insomnia, poor concentration, deteriorating educational performance, reluctance to work, predictor of posttraumatic stress and depressive symptoms, trauma-related mental health disorders, emotional disturbance, low mood, emotional exhaustion, fear, nervousness, sadness, fulfilling basic needs/services, boredom, lack of peer contact [4,6-14].

Although research on the implications of trauma-related events on children is expanding, studies on response and coping strategies remain scarce. Coping is an act (behavior) and effort (cognitive) to prevent or diminish threat, harm, and loss, or to reduce the distress that is often associated with stressful experiences [15]. We can say that stress exists whenever people face situations that exceed their ability to manage them [16]. Some theorists limit the concept of coping to voluntary responses [17]. Others include automatic and involuntary responses as well [18]. There are several taxonomies developed to identify coping strategies in the literature such as adaptive and maladaptive, problem-focused, emotion-focused, social support, cognitive reframing, and religious and spiritual coping [19,20]. Although the appropriateness of the strategies differs according to the type of stressor, strategies in general are considered functional if they succeed in diminishing or reducing the threat of the situation whether it is internal or external [20,21].

In Qatar, home isolation and social distancing regulations have been in effect since early March 2020. Taking into consideration the level of impact this virus has had and how it spreads, a national strategy was considered to mitigate transmission, by enforcing home isolation and social distancing regulations, following the recommendation of the National Crisis Committee led by the Ministry of Public Health.

Several measures were taken nationally, such as closing cinema theaters, malls, public transportation, children’s public playgrounds, gyms, and wedding venues, including those in hotels. In addition, the Ministry of Public Health called upon the public to avoid crowded places and postpone social gatherings. Entry into the country was suspended for all those wishing to travel from certain countries. For children and adolescents, the main measure involved suspending studies in school and switching to remote education from home [22-24]. Elderly people and those with chronic health conditions were advised to avoid going out unless necessary, as they are more susceptible to infections. Further precautions included taking care and applying simple preventive measures to protect against infection, such as washing one’s hands often, using hand sanitizers and masks, and maintaining a safe distance from people with symptoms of illness [22]. In addition, a COVID-19 contact center accessible around the clock was established for any reports or inquiries regarding the virus via a toll-free phone number (hotline: 16000) [22] .The National Crisis Committee set a 4-stage strategy for reopening the country, with stage 1 starting on June 15, 2020, and stage 4 to start in early September 2020. Each stage was expected to take 1 month, and increases in COVID-19 cases would be the key factor impacting the move from one stage to the next.

This study assessed how children and adolescents (7-18 years) reacted to changes in their daily routines and their behaviors due to the COVID-19 pandemic in Qatar. In particular, the study focused on the psychological influence of home isolation and social distancing on children and adolescents, and the strategies used to cope with the pandemic. In order to shed light on psychological impact, specific diagnoses were selected for this research: depression, general anxiety, separation anxiety, anger, and adjustment disorder.

## Methods

### Study Design

This is a cross-sectional study implemented on a national level to screen for a selected number of psychological changes in children and adolescents in Qatar during the COVID-19 crisis. While it is acknowledged that challenges may arise from the lack of standardized teleassessment methods, which can affect the means to identify psychological disorders in larger populations, it is important that measures be taken into consideration to address them, especially when some disorders have established procedures for assessment like in-person consultations [25].

An online questionnaire was introduced as a screening method to shed light on select psychological changes and their relation to adherence to national regulations and level of participant vulnerability to the virus. We also aimed to identify adaptive coping strategies practiced by children, adolescents, and their parents during the COVID-19 epidemic in Qatar. Sociodemographic characteristics were taken into consideration in relation to adherence to national regulations and vulnerability to the virus.

Adherence to national regulations and restrictions were used as an outcome measure since it represents a unified method for families to prevent the spread of COVID-19, as indicated by studies and guidelines by the World Health Organization. Since we focused on psychological changes in children and adolescents, it was also important to determine the extent to which this group is vulnerable to COVID-19, which was added as the second main outcome measure in the study. Approval from the ethical committee at the Primary Health Care Corporation (PHCC) was obtained to conduct this study.

This study tested the following hypotheses:

1. Is there an association between following the official regulations during home isolation and social distancing due to the COVID-19 pandemic with the sociodemographic characteristics of participants, final psychological diagnoses, and coping strategies used by children and adolescents?
2. Is there an association between the perceived level of vulnerability to SARS-CoV-2 during home isolation and social distancing due to the COVID-19 pandemic with sociodemographic characteristics of participants, final psychological diagnoses and coping strategies used by children and adolescents?
3. Is there any difference in the mean score of selected final psychological diagnoses and coping strategies used across the 3 levels of compliance with official regulations among children and adolescents during home isolation and social distancing due to the COVID-19 pandemic?
4. Is there any difference in the mean score of selected final psychological diagnoses and coping strategies used across the 3 perceived levels of vulnerability to SARS-CoV-2 among children and adolescents during home isolation and social distancing due to the COVID-19 pandemic?
5. Is there an association between the study population’s age and final psychological diagnoses?
6. Is there an association between the study population’s age and coping strategies?

### Study Population

All home-isolated children (7-12 years old) and adolescents (13-18 years old) registered at PHCC and living in Qatar during the COVID-19 pandemic (n=~170,000) were eligible to participate in the study. Children and adolescents with intellectual disadvantages (eg, being unable to communicate their thoughts) were excluded. While obtaining consent from parents to participate in the questionnaire, it was clarified that they must exclude children and adolescents who met the exclusion criterion.

PHCC is a government agency that provides primary health care services to the community in Qatar. Thus, it is the first point of contact between patients and health care providers.

### Sample Size and Technique

The research team shared the questionnaire with PHCC’s Health Information Management (HIM) department both in Arabic and English, and provided an explanatory introduction of the research and its objectives, including an approval form for consent to participate in the research. The HIM department developed an electronic questionnaire and consent form. It was sent to parents’ mobile phones on file at PHCC. Data was collected from 6608 participants from June 23 to July 18, 2020.

### Data Collection Tools

The approach of developing the questionnaire took into consideration several assessment tools to screen for anxiety (General Anxiety and Separation Anxiety and Spence Children’s Anxiety Scale [SCAS]), depression (Kutcher Adolescent Depression Scale [KADS]), and anger (Clinical Anger Scale [CAS]). Questions that were considered irrelevant to the COVID-19 pandemic and home isolation were disregarded.

For anxiety, the subscale calculation in SCAS was reviewed, and researchers disregarded questions that were meant to assess panic disorder/agoraphobia, obsessive compulsive disorder, social phobia, and anxiety of physical injury [26]. The local context of social distancing and home isolation enforcement by the National Crisis Committee was taken into consideration. Thus, questions on anxieties related to environments outside the home, such as schools, public spaces, public toilets, and transportations, were excluded. Questions related to general anxiety and separation anxiety were retained. We aimed to screen for the effects on children and adolescents caused by being separated from their parents during the pandemic, by taking into consideration its relation to the two outcome measures, vulnerability of children and adolescents to the virus as assessed by parents and older siblings leaving home on an occasional basis, and the level of adherence to regulations by parents and guardians who chose to leave home.

KADS was used as a basis for the questions used to screen for depression. Researchers customized the questions and simplified them for two main reasons: (1) to screen rather than diagnose and (2) to simplify them for comprehension by children by minimizing complex wording and assuring their suitability as addressed in the Diagnostic and Statistical Manual of Mental Disorders, 5th Edition (DSM-5). We refrained from using the diagnostic symptom of suicidal ideation, since the assessment was not performed in a clinical setting [27,28].

Although anger is not an explicit diagnosis in the DSM-5, several formally identified disorders reflect enduring and dysfunctional anger. The inclusion of anger in the questionnaire was to distinguish between aggression, indecisiveness, low concentration, distrust, and other symptoms related to depression and anxiety that the targeted population may express. The CAS is an objective, validated self-report measure of psychological symptoms, including anger, irritation, social interference, decision interference, and thinking interference [28-30].

According to the DSM-5, adjustment disorder is present when abnormal, emotional, or behavioral symptoms occur in response to stressful events such as a natural disaster (in this case, the event is a pandemic). Stressors may affect single individuals, an entire family, or larger community groups. Following the diagnostic criteria, questions were addressed to screen for emotional and behavioral symptoms developed within months of the start of the pandemic like change in sleep routine, boredom, wanting to be alone, stress and feeling upset, change in the rate of electronic device usage, and overall rate of daily activity. The symptoms identified do not meet criteria pertaining to other disorders collectively, where the stressor (home isolation) is considered an essential factor [28].

It is fundamental to identify coping subcategories since there are no specific strategies observed or a particular belief that can be reliably reported [31]. The literature argues that coping is considered an organizational construct, where it is practiced to manage stressful experiences. It is considered changeable and fixable according to the type of stressor and culture [31]. According to Aldwin [19], culture can affect the way stressors are reacted to and generally affect the choice of coping strategies adopted. Thus, 8 different practices applicable to the lifestyle of children and adolescents in Qatar were selected, with this research aiming to identify the level of adaption of these specific practices during the pandemic.

As far as categorizing coping practices, Edge and Sherwood [31] argue that a hierarchical view of coping can be useful to determine the link between the type of coping and adaptive coping to innumerable situation-specific responses. There is a strong correlation between adaptive coping practices and mental health; both general coping styles and specific strategies are significant in mediating the effects of stressful experiences and the development of psychological disorders. According to Edge and Sherwood [31], “a category is functionally homogenous to the extent it is defined so that all ways of coping it includes serve the same set of functions, ways of coping that are functionally homogenous should be able to be substituted for each other, thus are included within the same category”. The 8 adaptive strategies selected were categorized into 4 themes—spiritual/emotional, physical, social, and cognitive—as Likert-scale questions, for the purpose of measuring level of practice involvement and to identify how different strategies can influence, moderate, and reduce the effects of the pandemic.

The activities selected within those 4 subthemes were taken into consideration to shed light on how often participants prayed, were physically active, socialized with their parents, engaged in at-home activities, etc, and their correlation to the selected diagnoses to be screened for. As it is evident that physical activity and social engagement can reduce depression, and praying and emotional care can reduce symptoms of anger, we aimed to identify the level of practice associated with these coping strategies, in order to advise parents and guardians of the importance of these practices in reducing the symptoms of some psychological effects [19,20].

The T-scores of all tools previously mentioned were reviewed, and all questions were compared to the diagnostic criteria and symptoms of each diagnosis in the DSM-5. The time period of questionnaire distribution was also considered after regulations and mandates on home isolation and social distancing were enforced. Thus, we screened for persistent symptoms in children and adolescents, lasting at least 2 months. The scoring method was linked to the scoring system of the tools mentioned above and comparable to the severity levels of each diagnosis in the DSM-5 (mild, intermediate, or severe). The final scoring system of the questionnaire was standardized across all screening questions to ensure quality of screening and participants’ ability to answer them [28].

### Data Analysis

Frequencies with percentages were calculated for categorical variables, and mean (SD) values were calculated for discrete variables. Chi-square tests were used to check associations between categorical variables and the main variables of adherence to regulations and level of vulnerability to SARS-CoV-2. One-way ANOVA (analysis of variance) with post hoc analysis (Bonferroni) was used for continuous variables with adherence and vulnerability. Pearson correlation coefficients were calculated to assess the correlation among the final psychological diagnoses (anger, adjustment disorder, depression, general anxiety, and separation anxiety) and the 4 coping strategies (spiritual/emotional, social, physical, and cognitive). A *P* value of .05 (two-tailed) was considered to indicate statistical significance. SPSS (version 23.0, IBM Corp) was used for the statistical analysis.

## Results

The total number of participants in this study was 6608 children and adolescents aged 7-18 years (3.9% of the invited population of 170,000). The data collection period was from June 23 to July 18, 2020, during the period of home isolation and social distancing enforced due to the COVID-19 pandemic.

As shown in Table 1, the distribution of sociodemographic characteristics, final psychological diagnoses screened, and coping strategies used varied between children and adolescents. Adolescents comprised approximately one-third of the sample, and children made up two-thirds. Approximately half were male, and the other half were female. Nearly half of the participants were in primary school and about three-quarters came from middle-income families. Almost all the participants adhered to the official regulations during the period of home isolation and social distancing. A total of 69.1% (n=4568) of parents stated that their children were vulnerable to the virus, compared to 25% (n=1652) who expressed no vulnerability. Most psychological effects were mild among the participants, who used various coping strategies to manage psychological changes. The strategies included were spiritual/emotional, cognitive, social, and physical forms of coping.

Each main variable associated with psychological impacts and coping strategies was divided into 3 to 4 or more subcategories for an in-depth understanding of the main variables as shown in Tables 2 and 3.

**Table 1:** Frequency distribution of sociodemographic characteristics, final psychological diagnoses, and coping strategies used among children and adolescents during home isolation and social distancing due to the COVID-19 pandemic in Qatar in 2020 (N=6608).

Variable		Frequency, n (%)
**Language**		
	English	2983 (45.1)
	Arabic	3625 (54.9)
**Age (years)**		
	7-12	4148 (62.8)
	13-18	2460 (37.2)
**Gender**		
	Male	3354 (50.8)
	Female	3254 (49.2)
**Nationality**		
	Qatari	1374 (20.8)
	Non-Qatari Arab	2237 (33.9)
	South Asian	1907 (28.9)
	Other	1090 (16.5)
**Education**		
	Primary	3220 (48.7)
	Middle	1733 (26.2)
	Secondary	1355 (20.5)
	Completed secondary	300 (4.5)
**Income (QR^a^)**		
	Up to 5000	627 (9.5)
	5001-10,000	1443 (21.8)
	10,001-20,000	2034 (30.8)
	20,001-40,000	1448 (21.9)
	40,001-60,000	598 (9)
	≥60,001	458 (6.9)
**Adherence to official regulations**		
	Not following instructions	84 (1.3)
	Following instructions exactly	4981 (75.4)
	Doing more than what was instructed	1543 (23.4)
**Perceived level of vulnerability to SARS-CoV-2**		
	Not vulnerable at all	1652 (25)
	As vulnerable as an average person	4568 (69.1)
	Extremely vulnerable	388 (5.9)
**Anger**		
	None	4784 (72.4)
	Mild	1740 (26.3)
	Severe	84 (1.3)
**Adjustment disorder**		
	None	1349 (20.4)
	Mild	4396 (66.5)
	Moderate to severe	863 (13.1)
**Depression**		
	None	3760 (56.9)
	Mild	1927 (29.2)
	Moderate	661 (10)
	Severe	260 (3.9)
**General anxiety**		
	No	2646 (40)
	Mild	3285 (49.7)
	Intermediate	573 (8.7)
	Severe	104 (1.6)
**Separation anxiety**		
	None	5077 (76.8)
	Possible	1531 (23.2)
**Spiritual/emotional coping strategy**		
	Did not use	508 (7.7)
	Felt somewhat comfortable and maintained practice	2439 (36.9)
	Always felt comfortable and maintained practice	3661 (55.4)
**Cognitive coping strategy**		
	Did not use	701 (10.6)
	Felt somewhat comfortable and maintained practice	4220 (63.9)
	Always felt comfortable and maintained practice	1687 (25.5)
**Physical coping strategy**		
	Did not use	1931 (29.2)
	Felt somewhat comfortable and maintained practice	3539 (53.6)
	Always felt comfortable and maintained practice	1138 (17.2)
**Social coping strategy**		
	Did not use	379 (5.7)
	Felt somewhat comfortable and maintained practice	2260 (34.2)
	Always felt comfortable and maintained practice	3969 (60.1)

^a^QR: Qatari Rial. 1 USD=3.64 QR.

**Table 2:** Frequency distribution of symptoms related to each final psychological diagnosis among children and adolescents during home isolation and social distancing due to the COVID-19 pandemic in Qatar in 2020 (N=6608).

Main variable and subcategory			None of the time, n (%)		Rarely, n (%)		Some of the time, n (%)		Often, n (%)		All the time, n (%)
**Anger**											
	Became more aggressive	1697 (25.7)		1399 (21.2)		2622 (39.7)		703 (10.6)		187 (2.8)	
	Able to deal with one’s own emotion	1138 (17.2)		2057 (31.1)		2379 (36.0)		771 (11.7)		263 (4.0)	
	Thinking clearly	1496 (22.6)		2232 (33.8)		2079 (31.5)		641 (9.7)		160 (2.4)	
	Having no trust in what people say about the virus	1400 (21.2)		1540 (23.3)		2440 (36.9)		913 (13.8)		315 (4.8)	
	Having trouble making decisions	1280 (19.4)		1787 (27.0)		2425 (36.7)		794 (12.0)		322 (4.9)	
**Adjustment disorder**											
	Increased use of electronic devices	102 (1.5)		197 (3.0)		995 (15.1)		2334 (35.3)		2980 (45.1)	
	Being less active during the day	461 (7.0)		874 (13.2)		2070 (31.3)		2153 (32.6)		1050 (15.9)	
	Feeling relaxed	897 (13.6)		1817 (27.5)		2470 (37.4)		1054 (16.0)		370 (5.6)	
	Feeling upset all the time	1323 (20.0)		1987 (30.1)		2101 (31.8)		832 (12.6)		365 (5.5)	
	Feeling like being alone	2838 (42.9)		1454 (22.0)		1454 (22.0)		559 (8.5)		303 (4.6)	
	Feeling bored almost all the time	567 (8.6)		1233 (18.7)		2128 (32.2)		1538 (23.3)		1142 (17.3)	
	Sleeping during the day and staying up during the night	2175 (32.9)		1088 (16.5)		1385 (21.0)		1125 (17.0)		835 (12.6)	
**Depression**											
	Eating more fast food	1315 (19.9)		2139 (32.4)		2012 (30.4)		831 (12.6)		311 (4.7)	
	Feeling optimistic about the future	1715 (26.0)		1784 (27.0)		2063 (31.2)		698 (10.6)		348 (5.3)	
	Feeling hopeless	3328 (50.4)		1612 (24.4)		1189 (18.0)		324 (4.9)		155 (2.3)	
	Feeling tired	1323 (20.0)		1921 (29.1)		2297 (34.8)		738 (11.2)		329 (5.0)	
	Cannot concentrate when talking to people	1713 (25.9)		1920 (29.1)		1961 (29.7)		764 (11.6)		250 (3.8)	
	Not feeling like doing any sports or physical activities	1554 (23.5)		1393 (21.1)		1967 (29.8)		1092 (16.5)		602 (9.1)	
	Feeling that things will never be the same again	1662 (25.2)		1311 (19.8)		1826 (27.6)		1161 (17.6)		648 (9.8)	
**Generalized anxiety**											
	Wanting to be a better person	1715 (26.0)		1884 (28.5)		2024 (30.6)		630 (9.5)		355 (5.4)	
	Worrying about things	434 (6.6)		1148 (17.4)		2820 (42.7)		1513 (22.9)		693 (10.5)	
	Making sure that things are done correctly	328 (5.0)		865 (13.1)		1653 (25.0)		1819 (27.5)		1943 (29.4)	
	Having nightmares	2843 (43.0)		1994 (30.2)		1227 (18.6)		395 (6.0)		149 (2.3)	
	Thinking one did not do well in school	2269 (34.3)		1540 (23.3)		1732 (26.2)		737 (11.2)		330 (5.0)	
**Separation anxiety**											
	Feeling afraid of being alone at home	2373 (35.9)		1445 (21.9)		1244 (18.8)		746 (11.3)		800 (12.1)	
	Worrying about being close to people other than one’s own parents	1421 (21.5)		1496 (22.6)		1715 (26.0)		1206 (18.3)		770 (11.7)	
	Worrying that something will happen to him/her	2105 (31.9)		1854 (28.1)		1721 (26.0)		612 (9.3)		316 (4.8)	

**Table 3:** Frequency distribution of symptoms related to each coping strategy used among children and adolescents during home isolation and social distancing due to the COVID-19 pandemic in Qatar in 2020 (N=6608).

Main variable and subcategory			None of the time, n (%)		Rarely, n (%)		Some of the time, n (%)		Often, n (%)		All the time, n (%)
**Spiritual/emotional coping** **strategy**											
	Practicing prayer and religious worship more often	374 (5.7)		541 (8.2)		1460 (22.1)		2001 (30.3)		2232 (33.8)	
	Asking for support from people who care about them and understand his/her feelings (eg, parents, teachers, academic advisors, school administrative staff)	429 (6.5)		667 (10.1)		1404 (21.2)		1713 (25.9)		2395 (36.2)	
**Cognitive coping** **strategy**											
	Choosing to receive accurate information from parents and official channels instead of rumors from peers	165 (2.5)		304 (4.6)		916 (13.9)		1965 (29.7)		3258 (49.3)	
	Limiting using of electronic devices	1825 (27.6)		2221 (33.6)		1838 (27.8)		516 (7.8)		208 (3.1)	
**Social coping** **strategy**											
	Spending more time with family	89 (1.3)		330 (5.0)		958 (14.5)		1803 (27.3)		3428 (51.9)	
	Doing at-home activities with the family, such as cooking a meal or playing board games	326 (4.9)		857 (13.0)		2254 (34.1)		1939 (29.3)		1232 (18.6)	
**Physical coping** **strategy**											
	Doing more exercise	896 (13.6)		1947 (29.5)		2280 (34.5)		1006 (15.2)		479 (7.2)	
	Creating a sleep schedule	1160 (17.6)		1460 (22.1)		1805 (27.3)		1337 (20.2)		846 (12.8)	

Having understood the relative proportion of the study factors, the association of the study variables with the two main variables (ie, adherence to official regulations and perceived level vulnerability to the coronavirus) among the participants during home isolation and social distancing was tested; the results are presented in Tables 4 and 5.

The association between the different levels of adherence to official regulations and perceived levels of vulnerability to the virus and sociodemographic factors, clinical outcome, and coping strategies used by participants were shown to be statistically significant (*P*≤.05) in most cases.

As shown in Table 6, the difference in mean score of psychological changes and coping strategies used by the participants across the 3 levels of compliance with official regulations were observed. These results were clinically insignificant, except in a few cases. Table 6 shows that respondents who followed instructions reported lower anger and depression scores compared to those who did not follow them.

Unlike the previous two constructs, the mean percentage of general and separation anxiety continuously increased with increasing level of adherence to regulations. Similarly, the mean score for coping strategies used by participants increased significantly among those who followed official instructions. However, the mean score for adjustment disorder increased among the people who followed the exact instructions than those who did not (from 46.4% to 49.4%) and decreased slightly among those who did more than what was prescribed (48.3%).

Table 7 shows that the difference in mean score for all psychological changes and coping strategies used among the participants across the 3 levels of vulnerability to the coronavirus were statistically significant. There was no obvious trends between clinical outcomes and perceived vulnerability. However, the mean score increased with increases in the level of vulnerability in the case of anger and separation anxiety.

As the study population is actually very diverse in terms of age group, ranging from 7 to 18 years, Tables 8 and 9 show the differences in the relationship between different age categories and study outcomes. The differences across age groups were moderate in most cases.

**Table 4:** Association between adherence to official regulations and sociodemographic characteristics of participants, final psychological diagnoses, and coping strategies used by children and adolescents in Qatar in 2020 (N=6608).

Variable			Have not been following instructions, n (%)		Have been following instructions exactly, n (%)		Have been doing more than what was instructed, n (%)		*P* value	
**Language**										.15
	English	34 (40.5)		2222 (44.6)		727 (47.1)				
	Arabic	50 (59.5)		2759 (55.4)		816 (52.9)				
**Age**										.59
	7-12 years	54 (64.3)		3142 (63.1)		952 (61.7)				
	13-18 years	30 (35.7)		1839 (36.9)		591 (38.3)				
**Gender**										.35
	Male	45 (53.6)		2503 (50.3)		806 (52.2)				
	Female	39 (46.4)		2478 (49.7)		737 (47.8)				
**Nationality**										.001
	Qatari	38 (45.2)		1018 (20.4)		318 (20.6)				
	Non-Qatari Arab	15 (17.9)		1743 (35.0)		479 (31.0)				
	South Asian	22 (26.2)		1454 (29.2)		431 (27.9)				
	Other	9 (10.7)		766 (15.4)		315 (20.4)				
**Education**										.02
	Primary	36 (42.9)		2439 (49.0)		745 (48.3)				
	Middle	28 (33.3)		321 (26.5)		384 (24.9)				
	Secondary	13 (15.5)		1018 (20.4)		324 (21.0)				
	Completed secondary	7 (8.3)		203 (4.1)		90)5.8)				
**Income (QR^a^)**										.001
	Up to 5000	14 (16.7)		449 (9.0)		164 (10.0)				
	5001-10,000	12 (14.3)		1095 (22.0)		336 (21.8)				
	10,001-20,000	17 (20.2)		1551 (31.1)		466 (30.2)				
	20,001-40,000	11 (13.1)		1134 (22.8)		303 (19.6)				
	40,001-60,000	13 (15.5)		430 (8.6)		155 (10.0)				
	≥60,001	17 (20.2)		322 (6.5)		119 (7.7)				
**Anger**										.001
	No	53 (63.1)		3614 (72.6)		1117 (72.4)				
	Mild	26 (31)		1316 (26.4)		398 (25.8)				
	Severe	5 (6)		51 (1)		28 (1.8)				
**Adjustment disorder**										.01
	None	21 (25)		968 (19.4)		360 (23.3)				
	Mild	56 (66.7)		3362 (67.5)		978 (63.4)				
	Moderate to severe	7 (8.3)		651 (13.1)		205 (13.3)				
**Depression**										.04
	None	35 (41.7)		2831 (56.8)		894 (57.9)				
	Mild	38 (45.2)		1444 (29)		445 (28.8)				
	Moderate	7 (8.3)		512 (10.3)		142 (9.2)				
	Severe	4 (4.8)		194 (3.9)		62 (4)				
**General anxiety**										.76
	No	42 (50)		2006 (40.3)		598 (38.8)				
	Mild	32 (38.1)		2492 (50)		761 (49.3)				
	Intermediate	8 (9.5)		408 (8.2)		157 (10.2)				
	Severe	2 (2.4)		75 (1.5)		27 (1.7)				
**Separation anxiety**										.36
	None	70 (83.3)		3825 (76.8)		1182 (76.6)				
	Possible	14 (16.7)		1156 (23.2)		361 (23.4)				
**Spiritual/emotional coping strategy**										.001
	Did not use	16 (19)		377 (7.6)		115 (7.5)				
	Felt somewhat comfortable and maintained practice	38 (45.2)		1884 (37.8)		517 (33.5)				
	Always felt comfortable and maintained practice	30 (35.7)		2720 (54.6)		911 (59)				
**Cognitive coping strategy**										.001
	Did not use	31 (36.9)		516 (10.4)		154 (10.0)				
	Felt somewhat comfortable and maintained practice	44 (52.4)		3242 (65.1)		934 (60.5)				
	Always felt comfortable and maintained practice	9 (10.7)		1223 (24.6)		455 (29.5)				
**Physical coping strategy**										.01
	Did not use	36 (42.9)		1469 (29.5)		426 (27.6)				
	Felt somewhat comfortable and maintained practice	33 (39.3)		2691 (54)		815 (52.8)				
	Always felt comfortable and maintained practice	15 (17.9)		821 (16.5)		302 (19.6)				
**Social coping strategy**										.001
	Did not use	17 (20.2)		269 (5.4)		93 (6.0)				
	Felt somewhat comfortable and maintained practice	37 (44)		1752 (35.2)		471 (30.5)				
	Always felt comfortable and maintained practice	30 (35.7)		2960 (59.4)		979 (63.4)				

^a^QR: Qatari Rial. 1 USD=3.64 QR.

**Table 5:** Association between the perceived level of vulnerability to corona virus during home isolation and social distancing because of the COVID-19 pandemic with sociodemographic characteristics of participants, final psychological diagnoses and the coping strategies used among children and adolescents in Qatar in 2020 (N=6608).

Variable			Not vulnerable at all, n (%)		As vulnerable as an average person, n (%)		Extremely vulnerable, n (%)		*P* value	
**Language**										.001
	English	642 (38.9)		2027 (44.4)		314 (80.9)				
	Arabic	1010 (61.1)		2541 (55.6)		74 (19.1)				
**Age**										.01
	7-12 years	1022 (61.9)		2854 (62.5)		272 (70.1)				
	13-18 years	630 (38.1)		1714 (37.5)		116 (29.9)				
**Gender**										.58
	Male	849 (51.4)		2317 (50.7)		188 (48.5)				
	Female	803 (48.6)		2251 (49.3)		200 (51.5)				
**Nationality**										.001
	Qatari	412 (24.9)		945 (20.7)		17 (4.4)				
	Non-Qatari Arab	582 (35.2)		1592 (34.9)		63 (16.2)				
	South Asian	454 (27.5)		1239 (27.1)		214 (55.2)				
	Other	204 (12.3)		792 (17.3)		94 (24.2)				
**Education**										.001
	Primary	778 (47.1)		2254 (49.3)		188 (48.5)				
	Middle	495 (30.0)		1125 (24.6)		113 (29.1)				
	Secondary	305 (18.5)		980 (21.5)		70 (18.0)				
	Completed secondary	74 (4.5)		209 (4.6)		17 (4.4)				
**Income (QR^a^)**										.001
	Up to 5000	209 (12.7)		377 (8.3)		41 (10.6)				
	5001-10,000	375 (22.7)		936 (20.5)		132 (34.0)				
	10,001-20,000	478 (28.9)		1449 (31.7)		107 (27.6)				
	20,001-40,000	347 (21.0)		1023 (22.4)		78 (20.1)				
	40,001-60,000	130 (7.9)		447 (9.8)		21 (5.4)				
	≥60,001	113 (6.8)		336 (7.4)		9 (2.3)				
**Anger**										.02
	None	1246 (75.4)		3261 (71.4)		277 (71.4)				
	Mild	382 (23.1)		1252 (27.4)		106 (27.3)				
	Severe	24 (1.5)		55 (1.2)		5 (1.3)				
**Adjustment disorder**										.001
	None	428 (25.9)		825 (18.1)		96 (24.7)				
	Mild	1020 (61.7)		3124 (68.4)		252 (64.9)				
	Moderate to severe	204 (12.3)		619 (13.6)		40 (10.3)				
**Depression**										.001
	None	1013 (61.3)		2497 (54.7)		250 (64.4)				
	Mild	435 (26.3)		1410 (30.9)		82 (21.1)				
	Moderate	147 (8.9)		474 (10.4)		40 (10.3)				
	Severe	57 (3.5)		187 (4.1)		16 (4.1)				
**General anxiety**										.001
	None	732 (44.3)		1763 (38.6)		151 (38.9)				
	Mild	729 (44.1)		2347 (51.4)		209 (53.9)				
	Intermediate	161 (9.7)		393 (8.6)		19 (4.9)				
	Severe	30 (1.8)		65 (1.4)		9 (2.3)				
**Separation anxiety**										.01
	None	1286 (77.8)		3517 (77)		274 (70.6)				
	Possible	366 (22.2)		1051 (23)		114 (29.4)				
**Spiritual/emotional coping strategy**										.001
	Did not use	112 (6.8)		362 (7.9)		34 (8.8)				
	Felt somewhat comfortable and maintained practice	551 (33.4)		1759 (38.5)		129 (33.2)				
	Always felt comfortable and maintained practice	989 (59.9)		2447 (53.6)		225 (58)				
**Cognitive coping strategy**										.001
	Did not use	171 (10.4)		494 (10.8)		36 (9.3)				
	Felt somewhat comfortable and maintained practice	983 (59.5)		3022 (66.2)		215 (55.4)				
	Always felt comfortable and maintained practice	498 (30.1)		1052 (23)		137 (35.3)				
**Physical coping strategy**										.001
	Did not use	447 (27.1)		1392 (30.5)		92 (23.7)				
	Felt somewhat comfortable and maintained practice	826 (50)		2507 (54.9)		206 (53.1)				
	Always felt comfortable and maintained practice	379 (22.9)		669 (14.6)		90 (23.2)				
**Social coping strategy**										.002
	Did not use	107 (6.5)		253 (5.5)		19 (4.9)				
	Felt somewhat comfortable and maintained practice	515 (31.2)		1630 (35.7)		115 (29.6)				
	Always felt comfortable and maintained practice	1030 (62.3)		2685 (58.8)		254 (65.5)				

^a^QR: Qatari Rial. 1 USD=3.64 QR.

**Table 6:** The difference in the mean score of selected final psychological diagnoses and coping strategies used across the 3 levels of compliance with the official regulations among children and adolescents during home isolation and social distancing due to the COVID-19 pandemic in Qatar in 2020 (N=6608).

Variable	Total, mean (SD)	Have not been following instructions (n=84), mean (SD)	Have been following instructions exactly (n=4981), mean (SD)	Have been doing more than what was instructed (n=1543), mean (SD)	*P* value
Anger	37.3 (16.9)	41.1 (19.5)	37.5 (16.5)	36.7 (17.9)	.04^a^
Adjustment disorder	49.1 (17.7)	46.4 (18.2)	49.4 (17.5)	48.3 (18.3)	.05
Depression	35.7 (17.7)	37.8 (18.4)	35.9 (17.6)	35.0 (18.0)	.11
General anxiety	42 (15.6)	39.5 (16.9)	41.9 (15.4)	42.5 (16.0)	.15
Separation anxiety	37.1 (23.1)	30.7 (22.1)	36.9 (23.0)	38.0 (23.6)	.01^b^
Spiritual/emotional coping strategy	69.2 (24.4)	57.6 (27.4)	68.9 (24.1)	70.8 (24.8)	.001^c^
Cognitive coping strategy	55.5 (19.5)	40.3 (22.2)	55.2 (19.2)	57.3 (20.1)	.001^d^
Social coping strategy	70.9 (21.9)	58.3 (27.4)	70.6 (21.4)	72.7 (22.8)	.001^e^
Physical coping strategy	45.2 (24.0)	39.1 (24.8)	44.9 (23.9)	46.6 (24.2)	.01^f^

^a^Have not been following instructions vs have been following instructions exactly, *P*=.15; have not been following instructions vs have been doing more than what was instructed, *P*=.59; have been following instructions exactly vs have been doing more than what was instructed, *P*=.37.

^b^Have not been following instructions vs have been following instructions exactly, *P*=.04; have not been following instructions vs have been doing more than what was instructed, *P*=.01; have been following instructions exactly vs have been doing more than what was instructed, *P*=.26.

^c^Have not been following instructions vs have been following instructions exactly, *P*=.001; have not been following instructions vs have been doing more than what was instructed, *P*=.001; have been following instructions exactly vs have been doing more than what was instructed, *P*=.02.

^d^Have not been following instructions vs have been following instructions exactly, *P*=.001; have not been following instructions vs have been doing more than what was instructed, *P*=.001; have been following instructions exactly vs have been doing more than what was instructed, *P*=.001.

^e^Have not been following instructions vs have been following instructions exactly, *P*=.001; have not been following instructions vs have been doing more than what was instructed, *P*=.001; have been following instructions exactly vs have been doing more than what was instructed, *P*=.003.

^f^Have not been following instructions vs have been following instructions exactly, *P*=.08; have not been following instructions vs have been doing more than what was instructed, *P*=.01; have been following instructions exactly vs have been doing more than what was instructed, *P*=.05.

**Table 7:** The difference in the mean score of selected final psychological diagnoses and coping strategies used between the 3 perceived levels of vulnerability to the coronavirus among children and adolescents during home isolation and social distancing due to the COVID-19 pandemic in Qatar in 2020 (N=6608).

Variable	Total, mean (SD)	Not vulnerable at all (n=1652), mean (SD)	As vulnerable as an average person (n=4568), mean (SD)	Extremely vulnerable (n=388), mean (SD)	*P* value
Anger	37.3 (16.9)	34.9 (18.1)	38.1 (16.3)	38.5 (17.2)	.001^a^
Adjustment disorder	49.1 (17.7)	47.2 (18.8)	50.1 (17.2)	46.0 (17.8)	.001^b^
Depression	35.7 (17.7)	33.5 (18.2)	36.6 (17.4)	34.5 (18.0)	.001^c^
General anxiety	42 (15.6)	40.7 (16.9)	42.5 (15.0)	41.3 (15.7)	.001^d^
Separation anxiety	37.1 (23.1)	34.9 (24.1)	37.5 (22.7)	41.6 (22.8)	.001^e^
Spiritual/emotional coping strategy	69.2 (24.4)	70.9 (24.6)	68.5 (24.2)	70.5 (25.2)	.01^f^
Cognitive coping strategy	56.7 (20.5)	56.7 (20.5)	54.7 (19.0)	59.6 (20.8)	.001^g^
Social coping strategy	70.9 (21.9)	72.3 (22.6)	70.2 (21.5)	73.7 (22.4)	.001^h^
Physical coping strategy	45.2 (24.0)	48.0 (25.6)	43.9 (23.2)	48.8 (24.4)	.001^i^

^a^Not vulnerable at all vs as vulnerable as an average person, *P*=.001; not vulnerable at all vs extremely vulnerable, *P*=.001; as vulnerable as an average person vs extremely vulnerable *P*>.99.

^b^Not vulnerable at all vs as vulnerable as an average person, *P*=.001; not vulnerable at all vs extremely vulnerable, *P*=.71; as vulnerable as an average person vs extremely vulnerable *P*=.001.

^c^Not vulnerable at all vs as vulnerable as an average person, *P*=.001; not vulnerable at all vs extremely vulnerable, *P*=.98; as vulnerable as an average person vs extremely vulnerable *P*=.06.

^d^Not vulnerable at all vs as vulnerable as an average person, *P*=.001; not vulnerable at all vs extremely vulnerable, *P*>.99; as vulnerable as an average person vs extremely vulnerable *P*=.36.

^e^Not vulnerable at all vs as vulnerable as an average person, *P*=.001; not vulnerable at all vs extremely vulnerable, *P*=.001; as vulnerable as an average person vs extremely vulnerable *P*=.002.

^f^Not vulnerable at all vs as vulnerable as an average person, *P*=.002; not vulnerable at all vs extremely vulnerable, *P*>.99; as vulnerable as an average person vs extremely vulnerable *P*=.36.

^g^Not vulnerable at all vs as vulnerable as an average person, *P*=.001; not vulnerable at all vs extremely vulnerable, *P*=.02; as vulnerable as an average person vs extremely vulnerable *P*=.001.

^h^Not vulnerable at all vs as vulnerable as an average person, *P*=.002; not vulnerable at all vs extremely vulnerable, *P*=.73; as vulnerable as an average person vs extremely vulnerable *P*=.006.

^i^Not vulnerable at all vs as vulnerable as an average person, *P*=.001; not vulnerable at all vs extremely vulnerable, *P*>.99; as vulnerable as an average person vs extremely vulnerable *P*=.001.

**Table 8:** Association between age categories in the study population and the final psychological diagnoses (clinical outcomes) (N=6608).

Variable		Age category				*P* value
		7-12 years, n (%)	13-18 years, n (%)	7-18 years, n (%)		
**Anger**					.001	
	None	3007 (72.5)	1777 (72.2)	4784 (72.4)		
	Mild	1105 (26.6)	635 (25.8)	1740 (26.3)		
	Severe	36 (0.9)	48 (2)	84 (1.3)		
**Adjustment disorder**					<.001	
	None	915 (22.1)	434 (17.6)	1349 (20.4)		
	Mild	2797 (67.4)	1599 (65)	4396 (66.5)		
	Moderate to severe	436 (10.5)	427 (17.4)	863 (13.1)		
**Depression**					<.001	
	None	2435 (58.7)	1325 (53.9)	3760 (56.9)		
	Mild	1203 (29.0)	724 (24.4)	1927 (29.2)		
	Moderate	377 (9.1)	284 (11.5)	661 (10.0)		
	Severe	133 (3.2)	127 (5.2)	260 (3.9)		
**General anxiety**					<.001	
	No	1685 (40.6)	961 (39.1)	2646 (40.0)		
	Mild	2071 (49.9)	1214 (49.3)	3285 (49.7)		
	Intermediate	347 (8.4)	226 (9.2)	573 (8.7)		
	Severe	45 (1.1)	59 (2.4)	104 (1.6)		
**Separation anxiety**					<.001	
	None	2990 (72.1)	2087 (84.8)	5077 (76.8)		
	Possible	1158 (27.9)	373 (15.2)	1531 (23.2)		

**Table 9:** Association between age categories in the study population and the coping strategies used (N=6608).

Variable			Age category							*P* value
			7-12 years, n (%)		13-18 years, n (%)		7-18 years, n (%)			
**Spiritual/emotional coping strategy**									<.001	
	Did not use	259 (6.2)		249 (10.1)		508 (7.7)				
	Felt somewhat comfortable and maintained practice	1409 (34.0)		1030 (41.9)		2439 (36.9)				
	Always felt comfortable and maintained practice	2480 (59.8)		1181 (48.0)		3661 (55.4)				
**Cognitive coping strategy**									<.001	
	Did not use	360 (8.7)		341 (13.9)		701 (6.6)				
	Felt somewhat comfortable and maintained practice	2590 (62.4)		1630 (66.3)		4220 (63.9)				
	Always felt comfortable and maintained practice	1198 (28.9)		489 (19.9)		1687 (25.5)				
**Physical coping strategy**									.001	
	Did not use	1148 (27.7)		783 (31.8)		1931 (29.2)				
	Felt somewhat comfortable and maintained practice	2264 (54.6)		1275 (51.8)		3539 (53.6)				
	Always felt comfortable and maintained practice	736 (17.7)		402 (16.3)		1138 (17.2)				
**Social coping strategy**									<.001	
	Did not use	160 (3.9)		219 (8.9)		379 (5.7)				
	Felt somewhat comfortable and maintained practice	1265 (30.5)		995 (40.4)		2260 (34.2)				
	Always felt comfortable and maintained practice	2723 (65.6)		1246 (50.7)		3969 (60.1)				

## Discussion

### Principal Findings

In response to the COVID-19 pandemic, the education authorities in Qatar ordered a nationwide school closure as an emergency measure to prevent transmission of the infection per the mandates implemented by the National Crisis Committee and international procedures. Children and adolescents during the COVID-19 pandemic expressed psychological changes not limited to fears, uncertainties, and physical and social isolation, and may miss school for a prolonged period as a result. Understanding their reactions and emotions is essential to properly address their needs.

This study’s sample is representative of the Qatari population in a number of ways. The preliminary results indicated that there was no gender bias, making our sample an acceptable representative for the subsequent analysis of the outcomes. The educational level of the participants and their ages were in line with our expectations. This will assist in developing better reintegration strategies and treatment programs among students in schools. The nationality distribution of participants in this study aligned with the characteristics of the population distribution in Qatar, and this in turn indicates that this sample truly represents the population. The percentage of Qatari participants in this study was relatively higher than their percentage in the population, and this is mostly due to their positive response to participating in the study [32]. Furthermore, this distribution is considered a good representation of the population’s income level in Qatar.

Despite variation in sociodemographic characteristics, almost all the respondents (n=6524, 99%) took the required steps and followed the official regulations to safeguard themselves and their family members from the COVID-19 pandemic. This is indicative of the community’s awareness regarding the importance of following official guidelines, which are in line with the directives issued by the World Health Organization and the Ministry of Public Health in Qatar [33,34]. Due to the importance of following these instructions, we have relied on them to measure the outcomes of this study [35].

The parents of most participants identified reported that their children were either not vulnerable to the coronavirus or as vulnerable as an average person (n=6220, 94%). Adequate care and standard operating procedures need to be employed for the vulnerable group (n=388, 6%). This result positively coincides with a study in China, which reported that 75.2% of participants were worried about their family members contracting COVID-19 [2].

Clinical outcomes of this study had varying prevalence and levels of severity, which ranged from mild to severe. Higher prevalence was seen for adjustment disorder and general anxiety. These findings were partially supported by other studies [35,36]. In one study, 54% of participants rated the impact of the outbreak on their mental health as moderate to severe, with depressive symptoms and anxiety being the conditions most often stated [35].

A little more than one-quarter of the participants in this study reported anger as one of the psychological changes witnessed during home isolation. This was also observed in a study from the United States [37]. Mild adjustment disorder was prevalent in 66.5% (n=4396) of our sample; this may indicate that when children are out of school, they tend exhibit changes in behaviors and lifestyle pattern, are physically less active, and have more screen time and sleep disruptions [38].

In this study, 35.3% (n=2334) and 45.1% (n=2980) of participants increased use of electronic devices often or all the time, respectively. This was also seen in a study from China where internet and smartphone addiction had increased during the COVID-19 pandemic compared to the period before the pandemic [36]. Due to limited physical and social encounters with other individuals in the community such as friends and relatives, children increasingly reached out to new contacts and groups online, as seen by various other studies [8,12,36,39,40]. It can also be interpreted as causation for introducing virtual learning during home isolation and social distancing regulations [39]. In a study from China, 78.33% admitted that the main purpose of spending time online during the COVID-19 crisis was related to studies compared to 57.1% before the crisis [36]. This may be seen as a positive aspects of the current pandemic as indicated by related studies [41,42]. On the other hand, reports indicated that the distribution of child sexual exploitation materials online; cyberbullying; online risk-taking behaviors; potentially harmful content; inappropriate collection, use, and sharing of data; and ransomware appear to be on the increase [39,40]. Hence, it is important to know how children and adolescents deal with electronic materials and devices, especially during the COVID-19 pandemic, as more than 80% of this study’s participants reported an increase in the use of these devices more often or all the time. There is a need to support and empower children, providing them with safe online learning experiences and making online platforms safe and accessible to them [39].

About 30% (n=1960) of our participants reported disturbance in sleep schedule all the time or often. This was also seen in a study where approximately 21% of participants had sleeping disorders [12]. Another study showed that insomnia was prevalent during quarantine [37]. In our study, participants had nightmares in some of the time (n=1227, 18.6%), often (n=395, 6%), and all the time (n=149, 2.3%), compared to less than 15% in a study from China [12].

On the other hand, home isolation proved to have some positive outcomes during the pandemic. For example, many participants in this study and in other studies stated having more time for rest and relaxation [41]. Several factors can be attributed to this, such as the disappearance of external stressors (the absence of private and business appointments, guests, and business trips), strengthening a sense of community and cohesion. In addition, children troubled in school due to bullying or other stressors may embrace homeschooling and find it relieving since the main stressor in their everyday life no longer exists [35].

Similar to other studies [12,35,43], about 60% (n=3858) of children and adolescents in this study had mild to intermediate levels of general anxiety. Although children seem to be less vulnerable than adults to COVID-19, initial reports from China indicated that children and adolescents had been impacted psychologically and developed behavioral problems including anxiety [12]. In our study, the prevalence of general anxiety was relatively equal between children and adolescents, whereas anxiety was more prevalent among adolescents aged 13-18 years compared to children aged 7-12 years in another study [36]. More than 25% of that sample expressed worry, compared to 22.9% (n=1513) and 10.5% (n=693) of our participants who worried about things often and all the time, respectively [12].

On the other hand, separation anxiety has been common during the COVID-19 pandemic, even as most people practice home isolation and teaching is virtual [44]. The impact of having family members working or volunteering and parents going shopping for food and groceries, along with unclear social situations, could increase the level of separation anxiety in children and adolescents [44]. However, the nature of Qatari society and the presence of high levels of social cohesion and family stability could have helped reduce the emergence of separation anxiety in our sample. Regardless, social relations have primarily been limited to close family members, which may negatively impact children and adolescents, given the importance of other family members and peers for well-being [45,46].

During difficult times such as this pandemic, developing depression is highly expected. In line with other studies, about 40% (n=2588) of this study’s participants developed a mild to moderate form of depression [12,35-37,43]. A study from Canada during the severe acute respiratory syndrome (SARS) epidemic showed that 31.2% of 129 participants had depression due to quarantine measures [9]. In our study, some participants experienced loss of concentration when talking to others almost all the time (n=1014, 15.4%) or some of the time (1961, 29.7%). Similarly, children aged 6-18 years in a study from China were more likely to show inattention during the COVID-19 pandemic (*P*=.049) [12].

About 30% (n=1976) of our study’s population worried often or all the time about being near people other than own parents, with 26% (n=1715) experiencing this feeling some of the time. In line with this finding, another study indicated that clinging was one of the most severe psychological conditions demonstrated by children [12].

In terms of coping, participants in our study used different strategies to face psychological problems resulting from the pandemic. With the combined support of health care professionals, families, and other social connections, including friends and the school environment, children and adolescents can appropriately overcome and stabilize emotionally and physiologically [47-49].

Similar to other related studies, cognitive coping was used by our participants. Media and digital entertainment were used by families to relieve their children’s distress and address their concerns regarding the negative state they were facing. Obsessive requests for updates were identified among almost 30% of participants in one COVID-19 study [12,50]. Choosing to receive accurate information from parents and official channels instead of rumors from peers was practiced widely by about 80% (n=5223) of our study’s population all the time or often. This intervention was advised by a guideline developed for facing the COVID-19 and other crises [35,49].

The spiritual/emotional coping strategy was also used by participants. Close and open communication with children is the key to identifying any psychological issues and to comfort children during prolonged isolation [49,51]. Different types of emotional support from parents and avoidance of gossip about the pandemic can help to decrease the negative impacts of the crisis and in return lower clinical symptoms [50]. Similar to the sample of another study [52], the majority of participants in our study felt comfortable and maintained their practice of prayers and asked support from people who understand their feelings to relieve the negative conditions related to home isolation and social distancing. In addition, intrinsic religiousness has been suggested to help find meaning during a crisis; the relevance of affectivity shows the importance of a positive mood and attitude for stress-related growth [53]. The results of our study have shown that over 64% (n=4233) of the participants maintained their religious practices during isolation.

On a positive note, family cohesion in Qatar may have strengthened families to count on each other for support. Our study showed that more family time was allocated to children and adolescents, with more activities reported to have been practiced with siblings and parents. More time with caregivers can increase a sense of social support, which strengthens resiliency [41]. Thus, we see that social coping strategies represented a third option for the participants in this study in facing the pandemic. A guideline developed during early COVID-19 crisis support has recommended this intervention as an effective option [49].

Maintaining physical activity during home isolation was a critical aspect that was screened for. Adding physical activities during children’s and adolescents’ daily schedules can help maintain their health and fitness during health-related crises [54]. Our study showed that the use of physical activity to overcome negative psychological states was low, with 15.2% (n=1006) of participants reporting doing more exercise than before and only 7.2% (n=479) reporting doing exercise almost all the time. Fear of infection, hot weather, high humidity, and regulations limited movement outside the home, and relative shortage of infrastructure to support exercise and physical exertion may be the main factors behind this limitation. In contrast, a study from China indicated that higher levels of physical activities reached effective and very effective levels of practice (31.72% and 20.06%, respectively) [12].

### Strengths and Limitations

This study had various strengths. The results provide essential information to support decision making by education authorities concerned with the psychological state of students during the COVID-19 pandemic or in similar crises in the future. In addition, the age group of our sample is more prone to psychological problems than other age groups. The older groups have a better ability to deal with exceptional periods such as a pandemic; in contrast, younger groups may be less aware of the dangers and psychological changes associated with a pandemic. Additionally, the sample size was relatively large and representative, which increases the applicability and generalization of the results. The use of a well-designed questionnaire and standard statistical methods ensured the comparability of the results with other studies.

Several limitations also exist. First, the study was conducted on an age group that may be at risk of information bias. In order to improve the quality of data, the questionnaires were sent to their parents’ mobile phones so that they can help their children, especially those in the early childhood age. Specific parts of the questionnaire were answered by children or adolescents with the help of their parents. Second, due to the fear of COVID-19 spreading during the period of data collection, information was collected online, which may affect the quality of the data collected. Therefore, subsequent follow-up studies may be required in which data collection should be face to face. Third, the results of this study were influenced by the time period the research was conducted, the level of adherence to social distancing restrictions by family members, and the level of children and adolescents’ vulnerability to the virus.

### Conclusion and Recommendation

The purpose of this study was not to provide a final diagnosis of psychological disorders. Rather, it was to establish a sense of what children and adolescents have been facing during home isolation and social distancing in Qatar. Screening for psychological and social disruptions is of importance to develop reintegration strategies in schools, and for health care providers to assess and monitor behavioral changes and negative psychological impacts post–COVID-19. Children and adolescents experiencing higher levels of anxieties should be provided with extra attention during reintegration and transitional phases in schools, as they might express persistent worry, irritability, and perceive situations or people as threatening, which may negatively impact their performance in school and the ways in which they interact with their peers.

Although electronic devices and social media platforms may have lowered anxiety levels in some cases and promoted more interconnectedness during the COVID-19 pandemic among people on a community level, it is important to address how these platforms are used and how content is tailored to children and adolescents. Some platforms may not be well designed for children under 18 years of age; parents and caretakers should not only set boundaries on how much time each child or teenager spends on electronic devices, they should also navigate and audit content accessed online. Another aspect that must be taken into consideration by parents and caregivers is the importance of maintaining an active lifestyle for their children and not neglecting their physical health. This can be activated by following sports enthusiasts and athletes on social media platforms or following fitness channels broadcasting daily in-home exercises. Finally, continuity of care for children and adolescents should be ensured during all phases of the pandemic by parents and health care providers.

## Abbreviations

ANOVA: analysis of variance
CAS: Clinical Anger Scale
DSM-5: Diagnostic and Statistical Manual of Mental Disorders, 5th Edition
HIM: Health Information Management
KADS: Kutcher Adolescent Depression Scale
PHCC: Primary Health Care Corporation
SARS: severe acute respiratory syndrome
SCAS: Spence Children’s Anxiety Scale
